# Mucinous ovarian neoplasm – outcomes of a rare tumor

**DOI:** 10.1016/j.gore.2025.101996

**Published:** 2025-11-24

**Authors:** Bahareh Hamedi, Shilpa Mokshagundam, Antonio Lembo, S John Weroha, Michaela E. McGree, Amanda L. Tapia, Carrie L. Langstraat

**Affiliations:** a Department of Obstetrics and Gynecology, Division of General Gynecology, Mayo Clinic, Rochester, MN, USA; b Department of Obstetrics and Gynecology, Division of Gynecologic Oncology, Mayo Clinic, Rochester, MN, USA; c Department of Molecular Pharmacology and Experimental Therapeutics, Mayo Clinic, Rochester, MN, USA; d Department of Oncology, Mayo Clinic, Rochester, MN, USA; e Department of Quantitative Health Sciences, Division of Clinical Trials and Biostatistics, Mayo Clinic, Rochester, MN, USA

**Keywords:** Mucinous, Borderline, Adenocarcinoma, Survival

## Abstract

**Background:**

Mucinous epithelial ovarian (mEO) tumors are rare entities. In this study, we describe the management and outcomes of patients treated for mucinous borderline tumor and mucinous adenocarcinoma of the ovary.

**Methods:**

This was a retrospective review of patients with mEO tumors from 1988 − 2021 from a single institution database. Primary outcomes were disease recurrence and death within five years post-surgery, evaluated using Cox proportional hazards and Kaplan-Meier survival analysis.

**Results:**

We identified 262 patients with mEO tumors with a mean age of 54.9 years. One-hundred and forty-three patients (55 %) had borderline tumors and 119 (45 %) adenocarcinoma. Most patients underwent complete surgical resection (95 %).

Those with mucinous borderline tumors had favorable prognosis, with 5-year progression-free survival (PFS) and overall survival (OS) of 97%. Among patients with mucinous adenocarcinoma, 5-year PFS was 78% and 5-year OS was 80%. Fifty-seven patients with adenocarcinoma (48%) received adjuvant chemotherapy.

In univariate analysis, adenocarcinoma histology, residual disease, advanced FIGO stage (II-IV), and receipt of adjuvant chemotherapy were associated with recurrence and death within five years of surgery. While a considerable proportion of patients in both cohorts underwent gastrointestinal (GI) endoscopy, all findings were negative/benign.

**Conclusions:**

Mucinous tumors of the ovary have overall favorable survival outcomes. In this cohort, residual disease, histopathology, and receipt of adjuvant chemotherapy were associated with disease recurrence. In addition, GI workup was of limited utility. Further study is needed to clarify ideal adjuvant treatment and GI workup.

## Highlights

• Mucinous tumors of the ovary have overall favorable survival outcomes.
• In this cohort residual disease, histopathology, and receipt of adjuvant chemotherapy were associated with recurrence.
• GI workup was of limited utility.
• Further study is needed to clarify ideal adjuvant treatment and GI workup.

## 1 Introduction

Mucinous epithelial ovarian (mEO) tumors account for approximately 15 % of all primary ovarian tumors and are characterized by the presence of mucin-secreting epithelial cells. (Mills and Shanes, 2019 Dec) Mucinous epithelial ovarian tumors encompass a spectrum of histopathology, ranging from benign to borderline to malignant adenocarcinoma. While clinical presentation of mEO tumors varies, the majority of patients present with a large, unilateral mass. (Mills and Shanes, 2019 Dec, Boger-Megiddo and Weiss, 2005).

Borderline mEO tumors are also known as mucinous tumors of low malignant potential or atypical proliferative mucinous tumors. (Silverberg et al., 2004, Matsuo et al., 2019) Although the vast majority of borderline mEO tumors are stage I, approximately 10 % are associated with extra-ovarian spread. (Matsuo et al., 2019) Surgical staging of borderline mEO tumors generally includes resection of the primary borderline tumor with either unilateral salpingo-oophorectomy or ovarian cystectomy, cytologic washings, omentectomy, and peritoneal biopsies. Routine lymphadenectomy is not recommended as it does not affect overall survival (OS), and risk of nodal involvement is < 5 %. (Network and Cancer, 2024).

Meanwhile, mucinous adenocarcinoma is a rare subtype of epithelial ovarian cancer, accounting for 3 to 4 % of primary ovarian cancers. (Meagher et al., 2018 Sep, Seidman et al., 2003 Jul) In contrast to serous ovarian carcinomas, 65 to 80 % of mEO adenocarcinomas are diagnosed at an early stage and carry a favorable prognosis. (Meagher et al., 2018 Sep) However, women with stage III and IV mEO adenocarcinoma have poorer prognosis when compared to other subtypes of epithelial ovarian cancers, after matching for stage (Seidman et al., 2003 Jul).

Adding to the complexity of management is that adenocarcinoma of the stomach, small bowel, colon, appendix, pancreas, or breast can metastasize to the ovary and have similar histologic appearance to primary mEO. (Brown and Frumovitz, 2014 Jun) Differentiation of primary mEO adenocarcinoma from metastatic disease of gastrointestinal (GI) origin can inform patient counseling and treatment approach. Multiple strategies, including the evaluation of clinical characteristics and immunohistochemistry (IHC), have been utilized to help distinguish primary mEO adenocarcinoma from metastatic disease. (Brown and Frumovitz, 2014 Jun, Zhang Y, Li C, Luo S, Su Y, Gang X, Chu P, Zhang J, Wu H, Liu G. Retrospective Study of the Epidemiology, Pathology, and Therapeutic Management in Patients With Mucinous Ovarian Tumors. Technol Cancer Res Treat., 2020) While IHC often demonstrates cytokeratin 20 (CK20) positivity and cytokeratin 7 (CK7) negativity, additional markers such as caudal-type homeobox 2 (CDX2) cannot make the distinction between ovarian and GI origin. In addition, current National Comprehensive Cancer Network (NCCN) guidelines recommend upper and lower GI endoscopy in patients with mucinous ovarian neoplasms, although the clinical utility of this evaluation in diagnosis and treatment planning is unclear. (Network and Cancer, 2024).

There is limited literature describing practice patterns and outcomes for mucinous epithelial ovarian (mEO) tumors. Historically, management of mucinous tumors has been extrapolated from that of serous ovarian carcinoma. However, significant variation persists in surgical and oncologic approaches, including the extent of surgery (particularly lymphadenectomy), (Calma et al., 2022) the use of fertility-sparing versus radical surgery, (Kurnit and Frumovitz, 2022 Nov 7) performance of appendectomy or gastrointestinal evaluation, (Kurnit and Frumovitz, 2022 Nov 7) and decisions regarding adjuvant therapy. (Rajadevan et al., 2024 Aug) This variation largely reflects the rarity of the disease, historical treatment parallels with serous carcinoma, and emerging evidence that mucinous tumors exhibit distinct biological behavior and relative chemoresistance. Further investigation is warranted to clarify prognostic factors and optimize management strategies.

The primary aim of this retrospective study is to evaluate predictors of progression-free survival (PFS) and overall survival (OS) within five years after surgery in patients with borderline mEO tumors or mucinous adenocarcinoma who were diagnosed, treated, or followed at a single institution. Secondary aims include descriptive analyses of diagnostic workup, surgical management, and systemic therapy in this cohort.

## 2 Materials and methods

This was a retrospective, single-institution cohort study of all patients with borderline mEO tumors or mucinous adenocarcinoma who were evaluated at the Mayo Clinic in Rochester, MN between 1988 and 2021.

Patients were included if they met at least one of the following criteria: (1) presented with primary mEO tumors and underwent primary surgery with follow-up at our institution; (2) underwent primary mEO surgery at another institution, with relevant prior clinical documentation available for review, and subsequently received follow-up at our institution; or (3) presented with recurrent mEO tumors with previous relevant clinical documentation available. Patients without follow-up data were excluded.

Patient demographic data were extracted from the “Mayo Clinic Ovarian Mucinous Tumor Database”. This Database is an Excel file that includes the patients’ names, MRN, pathology diagnosis, and disease stage for individuals diagnosed with mucinous ovarian tumors. For each patient, relevant clinical characteristics were abstracted from the paper chart/electronic medical record including patient demographics, mucinous type (per pathology report as borderline vs adenocarcinoma), preoperative tumor markers, receipt of adjuvant chemotherapy, amount of residual disease at completion of surgery, International Federation of Gynecology and Obstetrics (FIGO) stage, completion of upper and lower GI endoscopy, and vital status.

Patient characteristics were compared by the type of mEO (borderline vs adenocarcinoma) using the T-test for age, Wilcoxon rank-sum test for body mass index (BMI), and chi-square or Fisher’s exact test for categorical variables. To assess PFS and OS in mEO tumors we used the Kaplan-Meier method, and differences by mEO type were tested using the log-rank test. Univariate Cox proportional hazard models were used to evaluate predictors of recurrence and death in mucinous adenocarcinoma within five years and summarized as hazard ratio (HR) and 95 % confidence interval (CI).

## 3 Results

### 3.1 Patient characteristics

We identified 262 patients with mEO tumors who met the inclusion criteria; 143 (54.6 %) had borderline histology and 119 (45.4 %) had adenocarcinoma. Patient characteristics are summarized in Table 1. When comparing the borderline and adenocarcinoma cohorts, the median preoperative CA-125 level was lower in patients with borderline histology (33.0) than in those with invasive adenocarcinoma (47.7). Regarding CEA levels, these were assessed in a limited number of cases, with a median preoperative level of 2.0 in the borderline cohort and 3.4 in the adenocarcinoma cohort. Omentectomy and lymphadenectomy were more frequently performed in patients with adenocarcinoma. Omentectomy was performed in 82 patients (57.3 %) with borderline tumors versus 101 patients (84.9 %) with adenocarcinoma, while lymphadenectomy was performed in 46 (32.2 %) and 81 (68.1 %) patients with borderline and adenocarcinoma, respectively.

**Table 1:** Patient characteristics by mucinous type (borderline vs adenocarcinoma).

**Characteristic**	**Total** **(N = 262)**	**Borderline** **(N = 143)**	**Adenocarcinoma** **(N = 119)**	**P**†
**Age at surgery (years), mean (SD)**	54.9 (15.4)	55.1 (15.6)	54.7 (15.2)	0.84
**BMI (kg/m^2^)**				0.49
N	233	127	106	
Median (IQR)	27.6 (23.6, 32.0)	27.1 (23.4, 32.9)	28.1 (24.7, 31.8)	
**Preoperative CA-125 (U/mL)**				0.001
N	218	121	97	
Median (IQR)	38.0 (19.0, 97.0)	33.0 (14.0, 77.0)	47.7 (23.0, 144.0)	
**Preoperative CEA (U/mL)**				0.25
N	45	28	17	
Median (IQR)	2.6 (1.1, 5.6)	2.0 (1.0, 4.1)	3.4 (1.9, 9.2)	
**Surgery, N (%)**				
Previous hysterectomy	17 (6.5)	11 (7.7)	6 (5.0)	0.39
Hysterectomy	177 (67.6)	86 (60.1)	91 (76.5)	0.005
Lymphadenectomy	127 (48.5)	46 (32.2)	81 (68.1)	<0.001
Previous appendectomy	28 (10.7)	15 (10.5)	13 (10.9)	0.91
Appendectomy	171 (65.3)	90 (62.9)	81 (68.1)	0.39
Small bowel resection	3 (1.1)	0 (0.0)	3 (2.5)	0.09
Large bowel resection	7 (2.7)	2 (1.4)	5 (4.2)	0.25
Omentectomy	183 (69.8)	82 (57.3)	101 (84.9)	<0.001
**Residual disease, N (%)**				0.006
No visible disease (RD0)	248 (94.7)	141 (98.6)	107 (89.9)	
≤10 mm	5 (1.9)	0 (0.0)	5 (4.2)	
>10 mm	5 (1.9)	1 (0.7)	4 (3.4)	
Unknown	4 (1.5)	1 (0.7)	3 (2.5)	
**FIGO stage, N (%)**				<0.001
I	229 (87.4)	139 (97.2)	90 (75.6)	
II/III/IV	32 (12.2)	4 (2.8)	28 (23.5)	
Unknown	1 (0.4)	0 (0.0)	1 (0.8)	
**Adjuvant chemotherapy,N (%)**				<0.001
No	196 (74.8)	140 (97.9)	56 (47.1)	
Yes	57 (21.8)	0 (0.0)	57 (47.9)	
Unknown	9 (3.4)	3 (2.1)	6 (5.0)	

Abbreviations: BMI, body mass index; FIGO, International Federation of Gynecology and Obstetrics; IQR, interquartile range; SD, standard deviation.

† T-test P value presented for age, Wilcoxon rank-sum P value presented for BMI, preoperative CA-125 and preoperative CEA, and chi-square or Fisher’s exact P value presented for categorical variables.

Of the patients with mucinous adenocarcinoma, 57 (47.9 %) received adjuvant chemotherapy. The majority of the patients who underwent adjuvant chemotherapy had a carboplatin-based regimen (50/57, 87.7 %), with 44 of the 50 patients receiving both carboplatin and paclitaxel. Other chemotherapy regimens included cisplatin-based (3/57, 5.3 %), oxaliplatin-based (1/57, 1.8 %), and 5-FU (1/57, 1.8 %) with two of unknown type (2/57, 3.5 %). In the patients that received adjuvant chemotherapy, 20 (35.1 %) recurred, 6 (10 %) were dead within 5 years, 9 (16 %) were alive with < 5 years of follow-up, and the remaining 22 (39 %) were alive and had 5 or more years of follow-up. Among the recurrences, 5 were stage I, 1 was stage II, 12 were stage III, and 2 were stage IV.

### 3.2 Recurrence location after primary treatment of mucinous ovarian tumors

Twenty-four patients with mEO tumors (9.2 % of the overall cohort) experienced a recurrence within five years after surgery. Recurrences were primarily seen in patients with adenocarcinoma, 21/119 (17.6 %), compared to only 3/143 (2.1 %) with borderline mEO. Eleven of the 24 patients had recurrences that involved multiple organs, which are summarized as 36 recurrence location events (Table 2). The most common recurrences were detected in the pelvis and upper abdomen. Distant metastasis (lung, pleura, and vertebrae) accounted for 25 % of the total events. Recurrence in pelvic and paraaortic lymph nodes were detected twice.

**Table 2:** Location of recurrence among the 24 patients with recurrence within 5 years after surgery (some patients had more than one recurrence locations).

**Recurrence location**	**N (%)**
Pelvis	10 (28)
Abdomen (including upper abdomen, spleen, liver, and pseudomyxoma peritonei)	15 (42)
Lymph nodes (pelvic, paraaortic, and mediastinal)	2 (5)
Thorax (lung/pleura)	8 (22)
Vertebra	1 (3)
Total number of recurrences	36

### 3.3 Progression-Free survival and predictors of recurrence

**Fig. 1:**
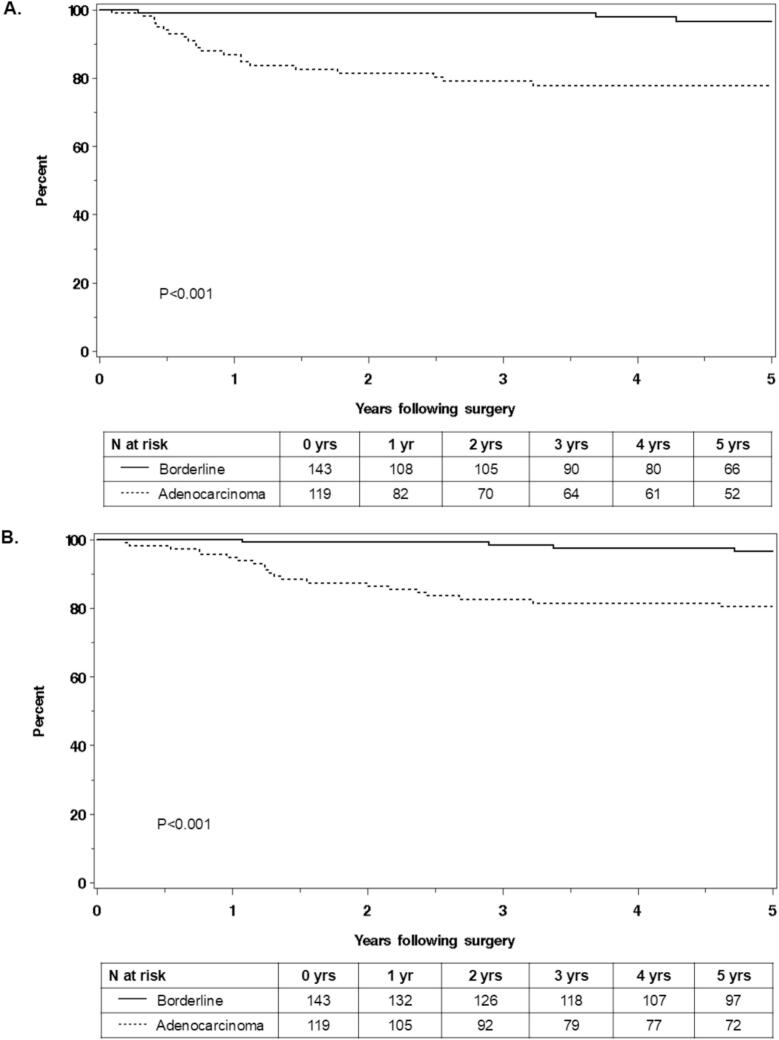
Kaplan-Meier survival curves for progression-free survival (A) and overall survival (B).

The 5-year PFS was significantly greater for patients with borderline tumors (97 %) vs those with adenocarcinoma (78 %) (log-rank p < 0.001) (Fig. 1). Within patients with adenocarcinoma, univariate analysis evaluated predictors for recurrence within five years after surgery (Table 3). We found no evidence of surgical factors such as performance of lymphadenectomy and appendectomy being associated with recurrence within five years after surgery. We observed that FIGO stage II-IV (HR 11.83, 95 % CI 4.74–29.54; p < 0.001), residual disease (HR 19.08, 95 % CI 6.78–53.64; p < 0.001), and receipt of adjuvant chemotherapy (HR 6.52, 95 % CI 1.92–22.14; p = 0.003) were associated with recurrence within five years after surgery.

**Table 3:** Univariate analysis for predictors of recurrence and death within 5 years after surgery among the patients with adenocarcinoma (N = 119).

**Characteristic**	**Recurrence within 5 years after surgery**		**Death within 5 years after surgery**	
	**N of events**	**HR (95 % CI)**	**N of events**	**HR (95 % CI)**
Age (years)	21	1.13 (0.84, 1.53)†	21	1.36 (1.02, 1.81)†
Lymphadenectomy at surgery				
No (N = 38)	7	Reference	7	Reference
Yes (N = 81)	14	0.79 (0.32, 1.97)	14	0.83 (0.34, 2.07)
Appendectomy (at surgery or previous)				
No (N = 25)	5	Reference	6	Reference
Yes (N = 94)	16	0.83 (0.31, 2.27)	15	0.68 (0.26, 1.74)
FIGO stage				
I (N = 90)	7	Reference	8	Reference
II/III/IV (N = 28)	14	11.83 (4.74, 29.54)	13	8.12 (3.34, 19.70)
Residual disease				
No (N = 107)	14	Reference	14	Reference
Yes (N = 9)	6	19.08 (6.78, 53.64)	6	13.14 (4.84, 35.65)
Adjuvant chemotherapy				
No (N = 56)	3	Reference	5	Reference
Yes (N = 57)	18	6.52 (1.92, 22.14)	15	3.33 (1.21, 9.16)

Abbreviations: CI, confidence interval; FIGO, International Federation of Gynecology and Obstetrics; HR, hazard ratio.

† Hazard ratio per 10-year increase in age.

### 3.4 Overall survival and predictors of death

The 5-year OS was 97 % in patients with mucinous borderline tumors and 80 % in patients with adenocarcinoma, and the survival curve for adenocarcinoma was significantly lower (log-rank p < 0.001). Within patients with adenocarcinoma, univariate analysis of predictors for death within five years after surgery (Table 3) did not reveal an association with surgical factors such as performance of lymphadenectomy and appendectomy. However, age at surgery (HR 1.36 per 10-year increase, 95 % CI 1.02–1.81; p = 0.04), FIGO stage II-IV (HR 8.12, 95 % CI 3.34–19.70; p < 0.001), residual disease (HR 13.14, 95 % CI 4.84–35.65; p < 0.001), and receipt of adjuvant chemotherapy (HR 3.33, 95 % CI 1.21–9.16; p = 0.02) were associated with death within five years after surgery.

### 3.5 Gastrointestinal endoscopy findings

We reviewed the completion and results of upper esophagogastroduodenoscopy (EGD), and colonoscopy within one year before or after diagnosis of mEO tumor. In the borderline cohort, 9 (6.3 %) had EGD and 29 (20.3 %) underwent colonoscopy, all with negative or benign findings. In the adenocarcinoma cohort, 25 (21.0 %) and 37 (31.1 %) patients underwent EGD and colonoscopy, respectively, and all patients had negative or benign findings (Table 4).

**Table 4:** Colonoscopy and EGD results by mucinous type (borderline vs adenocarcinoma).

**Characteristic, N (%)**	**Total** **(N = 262)**	**Borderline** **(N = 143)**	**Adenocarcinoma** **(N = 119)**	**P**†
Colonoscopy				0.003
No	153 (58.4)	97 (67.8)	56 (47.1)	
Yes	66 (25.2)	29 (20.3)	37 (31.1)	
Unknown	43 (16.4)	17 (11.9)	26 (21.8)	
Colonoscopy results				0.51
Negative	42/66 (63.6)	17/29 (58.6)	25/37 (67.6)	
Benign polyp	23/66 (34.8)	11/29 (37.9)	12/37 (32.4)	
Unknown	1/66 (1.5)	1/29 (3.4)	0/37 (0.0)	
EGD				<0.001
No	181 (69.1)	115 (80.4)	66 (55.5)	
Yes	34 (13.0)	9 (6.3)	25 (21.0)	
Unknown	47 (17.9)	19 (13.3)	28 (23.5)	
EGD results				0.66
Negative	25/34 (73.5)	6/9 (66.7)	19/25 (76.0)	
Benign findings	7/34 (20.6)	2/9 (22.2)	5/25 (20.0)	
Unknown	2/34 (5.9)	1/9 (11.1)	1/25 (4.0)	

Abbreviations: EGD, esophagogastroduodenoscopy.

† Chi-square or Fisher’s exact P value.

## 4 Discussion

This study is one of the largest retrospective reviews of a rare ovarian pathology known to date, spanning 34 years of clinical data. Patients were treated predominantly in a single center, thereby reducing between-practice heterogeneity. In this study, we quantified PFS and OS of mucinous borderline and adenocarcinoma ovarian tumors. Among patients with adenocarcinoma, we quantified associations between patient and treatment characteristics with recurrence and death within five years after surgery and identified the most common sites of tumor recurrence. Finally, we examined the result of upper and lower GI endoscopy in patients with mucinous ovarian tumors.

For patients with adenocarcinoma, factors associated with recurrence and death within five years of surgery include FIGO stage II-IV, residual disease, and receipt of adjuvant chemotherapy (likely reflecting higher-stage disease). This is consistent with existing literature which has demonstrated associations between invasive pattern, FIGO stage, and absence of benign or borderline component and survival. (Lee et al., 2022).

When considering adjuvant therapy, the majority of patients with mucinous ovarian adenocarcinoma in our study (50/57) received carboplatin-based chemotherapy. Due to the limited number of patients receiving alternative regimens, comparison of survival outcomes between different chemotherapy groups was not feasible. Nonetheless, there remains a lack of high-quality evidence to guide adjuvant therapy in this setting. Accumulating data suggest that mucinous epithelial ovarian carcinoma exhibits distinct biological behavior, a poorer response to standard ovarian-type regimens, and shares molecular features with mucinous gastrointestinal malignancies (Jones et al., 2019). Retrospective series have shown that treatment with GI-type regimens (e.g., oxaliplatin plus capecitabine or 5-fluorouracil) is associated with improved progression-free and overall survival compared to gynecologic-type chemotherapy (Kurnit et al., 2019 Dec). Moreover, given the challenges of patient accrual in dedicated trials—such as GOG 241, which closed early—no prospective randomized evidence has definitively established the superiority of GI-type therapy, and current recommendations remain cautious (Gore et al., 2019).Therefore, oncologists are likely to continue relying on retrospective data to inform chemotherapy decisions in this setting.

In addition, significant discrepancy exists in the literature regarding the origin of ovarian mucinous tumors. The GI tract is considered a potential primary origin of the mEO tumors. (Leen and Singh, 2012 Jul, Maeda et al., 2022 Mar 23, Mehmood and Khan, 2015) For this reason, the latest NCCN guideline recommends considering upper and lower GI endoscopy for new diagnoses of mEO tumors but no specific criteria for testing are defined. (Network and Cancer, 2024) Rather, clinical judgement should be used to minimize invasive procedures for patients with other clinical or diagnostic data supporting mEO rather than a GI neoplasm. Although none of the patients in our study had positive endoscopy findings, limitations are discussed below. Given that EGD and colonoscopy are invasive procedures, further research is warranted to establish clear patient selection criteria for GI evaluation.

The limitations of the present study include its descriptive and retrospective design, as well as the relatively small sample size, which limits the generalizability of the findings. Due to the retrospective nature of the study, patient records may not have been complete, which could introduce biases. Furthermore, the patients for this analysis were extracted from the Mayo Clinic Ovarian Mucinous Tumors Database. It is important to note that the database may not encompass patients whose tumors originated from a GI primary site. This exclusion could explain the negative results observed in our EGD and colonoscopy evaluations. In addition, recent advances in the pathological classification of mEO tumors have introduced new descriptors, such as the distinction between expansile and infiltrative types, as well as the use of IHC. However, such updated descriptors were not available in the pathology reports included in our cohort, as these were collected prior to the implementation of these newer diagnostic standards. Finally, while our study aimed to evaluate the role of tumor markers in mEO, CA-125 was the only marker consistently measured in our cohort (Table 1). Other commonly utilized tumor markers, such as carcinoembryonic antigen (CEA) and CA 19–9, were assessed in too few cases to allow meaningful analysis. We included CEA marker due to its relevance; however, the limited number of available cases precluded any firm conclusions. Therefore, the relationship between serum tumor markers and patient prognosis warrants further investigation in future studies with larger sample sizes to better elucidate their potential prognostic value.

In conclusion, mucinous tumors of the ovary are rare entities with overall favorable outcomes. In this cohort, however, patients with adenocarcinoma were more likely to have disease recurrence and die within five years after surgery compared to those with borderline disease. Additionally, in adenocarcinoma patients, need for adjuvant chemotherapy, presence of visible residual disease following cytoreduction, or advanced FIGO stage predicted greater risk for recurrence and death within 5 years. In addition, although NCCN guidelines currently recommend considering upper and lower GI endoscopy for patients with mucinous ovarian neoplasm, this evaluation is uncommon for patients in this study, and thus, conclusions cannot be drawn about its utility. Further study is needed to clarify ideal adjuvant treatment for this disease and understand the utility of GI evaluation.

## CRediT authorship contribution statement

**Bahareh Hamedi:** Writing – original draft, Methodology, Investigation. **Shilpa Mokshagundam:** Writing – review & editing, Resources. **Antonio Lembo:** Investigation, Data curation. **SJohn Weroha:** Writing – review & editing. **Michaela E. McGree:** Writing – review & editing, Formal analysis. **Amanda L. Tapia:** Writing – review & editing, Formal analysis. **Carrie L. Langstraat:** Writing – review & editing, Supervision, Project administration, Methodology, Conceptualization.

## Declaration of Competing Interest

The authors declare that they have no known competing financial interests or personal relationships that could have appeared to influence the work reported in this paper.
